# Di-μ-chlorido-bis­[(2-{(*E*)-[(2,3-dihy­droxy­prop­yl)imino]­meth­yl}phenolato)copper(II)] methanol monosolvate

**DOI:** 10.1107/S1600536811041481

**Published:** 2011-10-12

**Authors:** Yong Li

**Affiliations:** a Suzhou Vocational University, Suzhou 215104, People’s Republic of China

## Abstract

In the title compound, [Cu_2_Cl_2_(C_10_H_12_NO_3_)_2_]·CH_3_OH, each of the two Cu^II^ atoms is bound to two O and one N atoms of the bis-chelating monoanionic Schiff base and two bridging chloride ligands. The metal atoms each show a distorted square-pyramidal coordination geometry. Intra­molecular O—H⋯O hydrogen bonds occur. In the crystal, O—H⋯O hydrogen bonds join the components into a chain extending along the *a* axis.

## Related literature

For a uranyl complex of the same Schiff base ligand, see: Bharara *et al.* (2007 ▶). For two penta­nuclear manganese complexes of a similar Schiff base ligand, see: Yang *et al.* (2010 ▶).
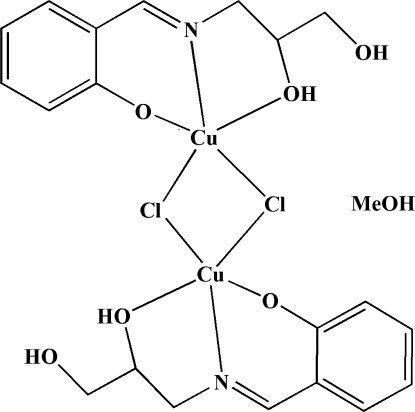


## Experimental

### 

#### Crystal data

[Cu_2_Cl_2_(C_10_H_12_NO_3_)_2_]·CH_4_O
*M* *_r_* = 618.43
Orthorhombic, 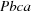

*a* = 15.490 (3) Å
*b* = 15.252 (3) Å
*c* = 19.951 (4) Å
*V* = 4713.6 (16) Å^3^
*Z* = 8
Mo *K*α radiation
μ = 2.08 mm^−1^
*T* = 113 K
0.14 × 0.12 × 0.08 mm

#### Data collection

Rigaku Saturn diffractometer
Absorption correction: multi-scan (*CrystalClear*; Rigaku/MSC, 2005 ▶) *T*_min_ = 0.760, *T*_max_ = 0.851
25866 measured reflections
4151 independent reflections
3727 reflections with *I* > 2σ(*I*)
*R*_int_ = 0.061

#### Refinement

*R*[*F* ^2^ > 2σ(*F* ^2^)] = 0.041
*wR*(*F* ^2^) = 0.092
*S* = 1.07
4151 reflections
313 parameters
H-atom parameters constrained
Δρ_max_ = 0.80 e Å^−3^
Δρ_min_ = −0.52 e Å^−3^

### 

Data collection: *CrystalClear* (Rigaku/MSC, 2005 ▶); cell refinement: *CrystalClear*; data reduction: *CrystalClear*; program(s) used to solve structure: *SHELXS97* (Sheldrick, 2008 ▶); program(s) used to refine structure: *SHELXL97* (Sheldrick, 2008 ▶); molecular graphics: *SHELXTL* (Sheldrick, 2008 ▶); software used to prepare material for publication: *SHELXTL* and *publCIF* (Westrip, 2010 ▶).

## Supplementary Material

Crystal structure: contains datablock(s) I, global. DOI: 10.1107/S1600536811041481/gk2407sup1.cif

Structure factors: contains datablock(s) I. DOI: 10.1107/S1600536811041481/gk2407Isup2.hkl

Additional supplementary materials:  crystallographic information; 3D view; checkCIF report

## Figures and Tables

**Table 1:** Hydrogen-bond geometry (Å, °)

*D*—H⋯*A*	*D*—H	H⋯*A*	*D*⋯*A*	*D*—H⋯*A*
O2—H2⋯O4	0.82	2.29	2.892 (4)	131
O3—H3⋯O7^i^	0.82	2.05	2.817 (6)	156
O3—H3⋯O6^i^	0.82	2.60	3.118 (6)	122
O5—H5⋯O7	0.82	1.88	2.567 (3)	141
O6—H6⋯O4^ii^	0.82	2.09	2.906 (4)	171
O7—H7*A*⋯O1	0.82	1.79	2.613 (5)	177

Symmetry codes: (i) 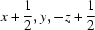; (ii) 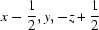.

**Fig. 1.**
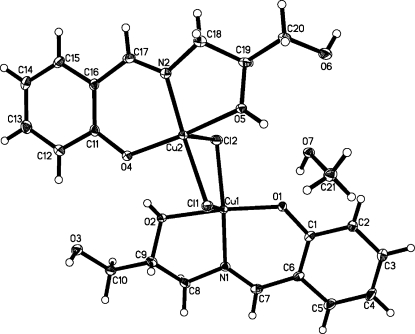
The molecular structure of the title complex with displacement ellipsoids shown at the 30% probability level.

**Fig. 2.**
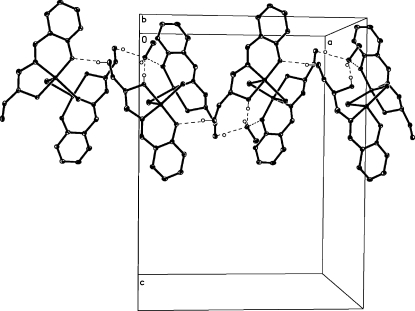
The hydrogen bonding interactions (dashed lines) between the crystal components components forming a chain extended along the a axis.

## supplementary crystallographic information

### Comment

Design and investigation of polynuclear transition metal complexes have received a great attention. The motivation in this field is justified not only by architectural beauty of the structures, but also by intellectual challenge of understanding the fundamental correlation between structures and magnetic properties (Yang *et al.*, 2010). Crucial to such efforts is the continuing development of new synthetic procedures of polynuclear transition metal species.

In order to synthesize new polynuclear paramagnetic clusters, we have recently begun to employ an asymmetric Schiff-base ligand, 3-(2-hydroxybenzylideneamino)-propane-1,2-diol, which contains a tetradentate {NO3} donor set and three hydroxyl groups that possess chelating and bridging capabilities. We believe that the four potential incorporable sites and multiple coordination modes will make the ligand a good candidate for the achievement of new polynuclear complexes. Here, we present the synthesis and structure of a new dinuclear copper complex, namely [Cu_2_(C_10_H_12_NO_3_)_2_Cl_2_].CH_3_OH (Scheme 1).

X-ray characterization of the title complex reveals that the dinuclear unit consists of two copper(II) atoms linked by two chloride ions. Each copper atom is coordinated by N-O-O atoms from a Schiff-base ligand and two chloride ions, providing a distorted square pyramidal environment (Fig. 1). Hydrogen bonds connect the complex into a chain extended along the a axis (Fig. 2).

### Experimental

3-(2-Hydroxybenzylideneamino)propane-1,2-diol (0.0384 g, 0.2 mmol) and NaOH (0.0080 g, 0.2 mmol) were dissolved in methanol (10 ml). A solution of CuCl_2_.2H_2_O (0.0341 g, 0.2 mmol) in methanol (10 ml) was added drop-wise into the previous mixture. The resulting solution was stirred at room temperature for two hours and then filtered. Green crystals suitable for X-ray crystallographic analysis were obtained by slow evaporation of the filtrate after one week.

### Refinement

All H atoms bound to C and O atoms were placed in calculated positions and treated as riding on their parent atoms, with C—H = 0.93-0.98 Å, *U*_iso_(H) = 1.2 or 1.5 *U*_eq_(C) and O—H = 0.82 Å, *U*_iso_(H) = 1.5 *U*_eq_(O).

### Crystal data

**Table:**

[Cu_2_Cl_2_(C_10_H_12_NO_3_)_2_]·CH_4_O	*F*(000) = 2528
*M**_r_* = 618.43	*D*_x_ = 1.743 Mg m^−^^3^
Orthorhombic, *P**b**c**a*	Mo *K*α radiation, λ = 0.71073 Å
Hall symbol: -P 2ac 2ab	Cell parameters from 9875 reflections
*a* = 15.490 (3) Å	θ = 2.1–27.9°
*b* = 15.252 (3) Å	µ = 2.08 mm^−^^1^
*c* = 19.951 (4) Å	*T* = 113 K
*V* = 4713.6 (16) Å^3^	Block, green
*Z* = 8	0.14 × 0.12 × 0.08 mm

### Data collection

**Table:**

Rigaku Saturn diffractometer	4151 independent reflections
Radiation source: rotating anode	3727 reflections with *I* > 2σ(*I*)
confocal	*R*_int_ = 0.061
ω scans	θ_max_ = 25.0°, θ_min_ = 2.1°
Absorption correction: multi-scan (*CrystalClear*; Rigaku/MSC, 2005)	*h* = −14→18
*T*_min_ = 0.760, *T*_max_ = 0.851	*k* = −18→16
25866 measured reflections	*l* = −23→23

### Refinement

**Table:**

Refinement on *F*^2^	Primary atom site location: structure-invariant direct methods
Least-squares matrix: full	Secondary atom site location: difference Fourier map
*R*[*F*^2^ > 2σ(*F*^2^)] = 0.041	Hydrogen site location: inferred from neighbouring sites
*wR*(*F*^2^) = 0.092	H-atom parameters constrained
*S* = 1.07	*w* = 1/[σ^2^(*F*_o_^2^) + (0.033*P*)^2^ + 12.2296*P*] where *P* = (*F*_o_^2^ + 2*F*_c_^2^)/3
4151 reflections	(Δ/σ)_max_ = 0.001
313 parameters	Δρ_max_ = 0.80 e Å^−^^3^
0 restraints	Δρ_min_ = −0.52 e Å^−^^3^

### Special details

**Table:**

Geometry. All esds (except the esd in the dihedral angle between two l.s. planes) are estimated using the full covariance matrix. The cell esds are taken into account individually in the estimation of esds in distances, angles and torsion angles; correlations between esds in cell parameters are only used when they are defined by crystal symmetry. An approximate (isotropic) treatment of cell esds is used for estimating esds involving l.s. planes.
Refinement. Refinement of *F*^2^ against ALL reflections. The weighted *R*-factor *wR* and goodness of fit *S* are based on *F*^2^, conventional *R*-factors *R* are based on *F*, with *F* set to zero for negative *F*^2^. The threshold expression of *F*^2^ > σ(*F*^2^) is used only for calculating *R*-factors(gt) *etc*. and is not relevant to the choice of reflections for refinement. *R*-factors based on *F*^2^ are statistically about twice as large as those based on *F*, and *R*- factors based on ALL data will be even larger.

### Fractional atomic coordinates and isotropic or equivalent isotropic displacement parameters (Å^2^)

**Table:**

	*x*	*y*	*z*	*U*_iso_*/*U*_eq_	
Cu1	0.17247 (3)	0.36330 (3)	0.23298 (2)	0.01406 (13)	
Cu2	0.10400 (3)	0.55924 (3)	0.29704 (2)	0.01452 (13)	
Cl1	0.21021 (6)	0.53619 (6)	0.21971 (4)	0.0182 (2)	
Cl2	0.04687 (6)	0.39157 (6)	0.28827 (5)	0.0238 (2)	
O1	0.11758 (17)	0.35442 (16)	0.14838 (12)	0.0180 (6)	
O2	0.24115 (16)	0.36996 (16)	0.31914 (12)	0.0163 (5)	
H2	0.2355	0.3985	0.3537	0.024*	
O3	0.37759 (19)	0.40175 (17)	0.41635 (13)	0.0237 (6)	
H3	0.4112	0.4411	0.4061	0.036*	
O4	0.17995 (17)	0.53598 (17)	0.37053 (12)	0.0196 (6)	
O5	0.02226 (17)	0.59528 (17)	0.22427 (12)	0.0187 (6)	
H5	0.0213	0.5865	0.1837	0.028*	
O6	−0.14092 (18)	0.58858 (19)	0.14549 (15)	0.0277 (7)	
H6	−0.1923	0.5754	0.1456	0.042*	
N1	0.2719 (2)	0.29782 (19)	0.20265 (15)	0.0168 (7)	
N2	0.0151 (2)	0.6110 (2)	0.35199 (16)	0.0209 (7)	
C1	0.1449 (3)	0.3126 (2)	0.09378 (18)	0.0172 (8)	
C2	0.0904 (3)	0.3119 (2)	0.03777 (18)	0.0185 (8)	
H2A	0.0372	0.3400	0.0399	0.022*	
C3	0.1147 (3)	0.2701 (2)	−0.02049 (19)	0.0231 (9)	
H3A	0.0781	0.2714	−0.0574	0.028*	
C4	0.1927 (3)	0.2260 (3)	−0.0249 (2)	0.0277 (10)	
H4	0.2082	0.1971	−0.0641	0.033*	
C5	0.2465 (3)	0.2259 (3)	0.02955 (19)	0.0264 (9)	
H5A	0.2991	0.1967	0.0266	0.032*	
C6	0.2247 (3)	0.2685 (2)	0.08995 (18)	0.0181 (8)	
C7	0.2841 (3)	0.2643 (2)	0.14478 (19)	0.0209 (8)	
H7	0.3359	0.2348	0.1377	0.025*	
C8	0.3367 (2)	0.2832 (2)	0.25562 (19)	0.0206 (8)	
H8A	0.3939	0.2801	0.2360	0.025*	
H8B	0.3251	0.2282	0.2783	0.025*	
C9	0.3326 (3)	0.3577 (2)	0.30512 (19)	0.0206 (8)	
H9	0.3568	0.4110	0.2851	0.025*	
C10	0.3819 (3)	0.3342 (2)	0.36812 (19)	0.0197 (8)	
H10A	0.3582	0.2807	0.3870	0.024*	
H10B	0.4419	0.3232	0.3568	0.024*	
C11	0.1593 (3)	0.5444 (2)	0.43551 (19)	0.0182 (8)	
C12	0.2192 (3)	0.5162 (3)	0.48352 (19)	0.0236 (9)	
H12	0.2716	0.4928	0.4694	0.028*	
C13	0.2021 (3)	0.5225 (3)	0.5512 (2)	0.0269 (9)	
H13	0.2429	0.5028	0.5820	0.032*	
C14	0.1249 (3)	0.5577 (3)	0.5742 (2)	0.0244 (9)	
H14	0.1137	0.5618	0.6199	0.029*	
C15	0.0658 (3)	0.5862 (2)	0.52858 (19)	0.0220 (9)	
H15	0.0141	0.6098	0.5438	0.026*	
C16	0.0809 (3)	0.5810 (2)	0.45905 (19)	0.0188 (8)	
C17	0.0135 (3)	0.6114 (2)	0.41642 (19)	0.0209 (8)	
H17	−0.0359	0.6333	0.4369	0.025*	
C18	−0.0575 (3)	0.6484 (3)	0.3144 (2)	0.0269 (9)	
H18A	−0.0474	0.7102	0.3060	0.032*	
H18B	−0.1104	0.6427	0.3400	0.032*	
C19	−0.0652 (2)	0.5999 (3)	0.2495 (2)	0.0220 (9)	
H19	−0.0868	0.5405	0.2580	0.026*	
C20	−0.1234 (3)	0.6453 (2)	0.20004 (19)	0.0202 (8)	
H20A	−0.1770	0.6616	0.2218	0.024*	
H20B	−0.0958	0.6983	0.1839	0.024*	
C21	0.0663 (3)	0.5456 (3)	0.0691 (2)	0.0254 (9)	
H21A	0.0262	0.5892	0.0538	0.038*	
H21B	0.0817	0.5079	0.0324	0.038*	
H21C	0.1172	0.5738	0.0861	0.038*	
O7	0.02716 (17)	0.49461 (16)	0.12123 (12)	0.0186 (6)	
H7A	0.0573	0.4517	0.1292	0.028*	

### Atomic displacement parameters (Å^2^)

**Table:**

	*U*^11^	*U*^22^	*U*^33^	*U*^12^	*U*^13^	*U*^23^
Cu1	0.0131 (2)	0.0143 (2)	0.0148 (2)	0.00271 (17)	−0.00061 (18)	−0.00130 (17)
Cu2	0.0124 (2)	0.0157 (2)	0.0154 (2)	0.00183 (17)	0.00014 (18)	−0.00179 (17)
Cl1	0.0163 (5)	0.0192 (4)	0.0190 (4)	0.0003 (3)	0.0018 (4)	−0.0014 (3)
Cl2	0.0189 (5)	0.0237 (5)	0.0288 (5)	0.0029 (4)	0.0013 (4)	−0.0048 (4)
O1	0.0180 (14)	0.0212 (13)	0.0147 (13)	0.0045 (11)	−0.0023 (11)	−0.0056 (10)
O2	0.0144 (13)	0.0196 (13)	0.0147 (12)	0.0020 (10)	0.0007 (11)	−0.0031 (10)
O3	0.0212 (16)	0.0245 (14)	0.0255 (15)	−0.0028 (12)	0.0028 (12)	−0.0068 (12)
O4	0.0158 (14)	0.0275 (14)	0.0153 (13)	0.0022 (11)	0.0003 (11)	−0.0022 (11)
O5	0.0159 (14)	0.0261 (14)	0.0143 (13)	0.0037 (11)	0.0003 (11)	−0.0014 (11)
O6	0.0163 (15)	0.0341 (16)	0.0329 (16)	0.0005 (12)	−0.0020 (13)	−0.0101 (13)
N1	0.0159 (16)	0.0160 (15)	0.0185 (16)	0.0017 (13)	−0.0001 (13)	−0.0007 (13)
N2	0.0197 (18)	0.0240 (17)	0.0190 (17)	0.0049 (14)	−0.0013 (14)	−0.0028 (13)
C1	0.022 (2)	0.0135 (18)	0.0161 (19)	−0.0021 (15)	0.0044 (16)	−0.0011 (14)
C2	0.021 (2)	0.0177 (18)	0.0169 (19)	0.0003 (15)	−0.0005 (16)	0.0024 (15)
C3	0.029 (2)	0.022 (2)	0.019 (2)	0.0005 (17)	−0.0017 (18)	0.0005 (16)
C4	0.038 (3)	0.031 (2)	0.015 (2)	0.0042 (19)	0.0027 (19)	−0.0068 (16)
C5	0.029 (2)	0.026 (2)	0.024 (2)	0.0060 (18)	0.0050 (19)	−0.0062 (17)
C6	0.021 (2)	0.0141 (17)	0.0192 (19)	0.0008 (15)	0.0047 (16)	−0.0005 (15)
C7	0.019 (2)	0.0175 (18)	0.026 (2)	0.0028 (15)	0.0041 (17)	−0.0018 (16)
C8	0.0151 (19)	0.0224 (19)	0.024 (2)	0.0034 (16)	−0.0024 (17)	−0.0021 (16)
C9	0.017 (2)	0.0217 (19)	0.023 (2)	−0.0020 (15)	0.0009 (17)	0.0009 (16)
C10	0.016 (2)	0.0205 (19)	0.022 (2)	0.0016 (16)	0.0008 (16)	−0.0011 (16)
C11	0.018 (2)	0.0185 (18)	0.0183 (19)	−0.0053 (15)	−0.0019 (16)	−0.0017 (15)
C12	0.019 (2)	0.030 (2)	0.022 (2)	0.0025 (17)	−0.0019 (17)	−0.0017 (17)
C13	0.028 (2)	0.032 (2)	0.020 (2)	0.0007 (19)	−0.0056 (18)	0.0027 (18)
C14	0.031 (2)	0.026 (2)	0.0158 (19)	−0.0057 (18)	0.0008 (18)	−0.0036 (16)
C15	0.024 (2)	0.024 (2)	0.019 (2)	−0.0020 (17)	0.0044 (17)	−0.0046 (16)
C16	0.019 (2)	0.0183 (18)	0.019 (2)	−0.0045 (15)	−0.0008 (16)	−0.0042 (15)
C17	0.017 (2)	0.0204 (19)	0.026 (2)	0.0021 (15)	0.0040 (17)	−0.0043 (16)
C18	0.023 (2)	0.030 (2)	0.027 (2)	0.0074 (18)	−0.0018 (18)	−0.0049 (18)
C19	0.014 (2)	0.027 (2)	0.025 (2)	0.0037 (16)	0.0029 (17)	−0.0007 (17)
C20	0.0152 (19)	0.0227 (19)	0.023 (2)	0.0064 (16)	0.0023 (17)	−0.0006 (16)
C21	0.026 (2)	0.028 (2)	0.022 (2)	−0.0009 (18)	0.0041 (18)	0.0042 (17)
O7	0.0172 (14)	0.0201 (13)	0.0184 (13)	0.0051 (11)	0.0015 (11)	0.0021 (11)

### Geometric parameters (Å, °)

**Table:**

Cu1—O1	1.895 (2)	C6—C7	1.431 (5)
Cu1—N1	1.933 (3)	C7—H7	0.9300
Cu1—O2	2.024 (2)	C8—C9	1.507 (5)
Cu1—Cl2	2.2777 (11)	C8—H8A	0.9700
Cu1—Cl1	2.7139 (11)	C8—H8B	0.9700
Cu2—O4	1.913 (3)	C9—C10	1.514 (5)
Cu2—N2	1.929 (3)	C9—H9	0.9800
Cu2—O5	2.003 (3)	C10—H10A	0.9700
Cu2—Cl1	2.2826 (10)	C10—H10B	0.9700
Cu2—Cl2	2.7118 (12)	C11—C12	1.401 (5)
O1—C1	1.331 (4)	C11—C16	1.417 (5)
O2—C9	1.457 (5)	C12—C13	1.380 (6)
O2—H2	0.8200	C12—H12	0.9300
O3—C10	1.412 (4)	C13—C14	1.389 (6)
O3—H3	0.8200	C13—H13	0.9300
O4—C11	1.341 (4)	C14—C15	1.361 (6)
O5—C19	1.447 (4)	C14—H14	0.9300
O5—H5	0.8200	C15—C16	1.409 (5)
O6—C20	1.416 (5)	C15—H15	0.9300
O6—H6	0.8200	C16—C17	1.424 (5)
N1—C7	1.277 (5)	C17—H17	0.9300
N1—C8	1.474 (5)	C18—C19	1.496 (5)
N2—C17	1.286 (5)	C18—H18A	0.9700
N2—C18	1.468 (5)	C18—H18B	0.9700
C1—C2	1.400 (5)	C19—C20	1.505 (5)
C1—C6	1.411 (5)	C19—H19	0.9800
C2—C3	1.378 (5)	C20—H20A	0.9700
C2—H2A	0.9300	C20—H20B	0.9700
C3—C4	1.386 (6)	C21—O7	1.434 (4)
C3—H3A	0.9300	C21—H21A	0.9600
C4—C5	1.369 (6)	C21—H21B	0.9600
C4—H4	0.9300	C21—H21C	0.9600
C5—C6	1.410 (5)	O7—H7A	0.8200
C5—H5A	0.9300		
			
O1—Cu1—N1	92.39 (12)	N1—C8—H8B	109.9
O1—Cu1—O2	174.84 (11)	C9—C8—H8B	109.9
N1—Cu1—O2	82.71 (11)	H8A—C8—H8B	108.3
O1—Cu1—Cl2	93.54 (8)	O2—C9—C8	105.3 (3)
N1—Cu1—Cl2	158.44 (9)	O2—C9—C10	111.2 (3)
O2—Cu1—Cl2	91.61 (8)	C8—C9—C10	110.2 (3)
O1—Cu1—Cl1	94.54 (8)	O2—C9—H9	110.0
N1—Cu1—Cl1	107.46 (9)	C8—C9—H9	110.0
O2—Cu1—Cl1	85.46 (7)	C10—C9—H9	110.0
Cl2—Cu1—Cl1	92.71 (3)	O3—C10—C9	111.6 (3)
O4—Cu2—N2	94.55 (12)	O3—C10—H10A	109.3
O4—Cu2—O5	174.28 (11)	C9—C10—H10A	109.3
N2—Cu2—O5	81.28 (12)	O3—C10—H10B	109.3
O4—Cu2—Cl1	92.65 (8)	C9—C10—H10B	109.3
N2—Cu2—Cl1	164.07 (10)	H10A—C10—H10B	108.0
O5—Cu2—Cl1	90.45 (8)	O4—C11—C12	118.3 (3)
O4—Cu2—Cl2	94.32 (8)	O4—C11—C16	124.2 (3)
N2—Cu2—Cl2	100.93 (10)	C12—C11—C16	117.5 (3)
O5—Cu2—Cl2	90.33 (8)	C13—C12—C11	121.4 (4)
Cl1—Cu2—Cl2	92.66 (3)	C13—C12—H12	119.3
Cu2—Cl1—Cu1	85.90 (3)	C11—C12—H12	119.3
Cu1—Cl2—Cu2	86.04 (4)	C12—C13—C14	120.9 (4)
C1—O1—Cu1	128.4 (2)	C12—C13—H13	119.5
C9—O2—Cu1	110.0 (2)	C14—C13—H13	119.5
C9—O2—H2	109.5	C15—C14—C13	118.8 (4)
Cu1—O2—H2	133.2	C15—C14—H14	120.6
C10—O3—H3	109.5	C13—C14—H14	120.6
C11—O4—Cu2	125.2 (2)	C14—C15—C16	121.9 (4)
C19—O5—Cu2	110.7 (2)	C14—C15—H15	119.0
C19—O5—H5	109.5	C16—C15—H15	119.0
Cu2—O5—H5	133.0	C15—C16—C11	119.4 (4)
C20—O6—H6	109.5	C15—C16—C17	116.7 (4)
C7—N1—C8	119.1 (3)	C11—C16—C17	124.0 (3)
C7—N1—Cu1	127.5 (3)	N2—C17—C16	125.6 (4)
C8—N1—Cu1	113.3 (2)	N2—C17—H17	117.2
C17—N2—C18	119.6 (3)	C16—C17—H17	117.2
C17—N2—Cu2	125.7 (3)	N2—C18—C19	108.1 (3)
C18—N2—Cu2	114.6 (2)	N2—C18—H18A	110.1
O1—C1—C2	117.8 (3)	C19—C18—H18A	110.1
O1—C1—C6	123.4 (3)	N2—C18—H18B	110.1
C2—C1—C6	118.8 (3)	C19—C18—H18B	110.1
C3—C2—C1	120.8 (4)	H18A—C18—H18B	108.4
C3—C2—H2A	119.6	O5—C19—C18	104.5 (3)
C1—C2—H2A	119.6	O5—C19—C20	110.8 (3)
C2—C3—C4	121.0 (4)	C18—C19—C20	112.8 (3)
C2—C3—H3A	119.5	O5—C19—H19	109.5
C4—C3—H3A	119.5	C18—C19—H19	109.5
C5—C4—C3	118.9 (4)	C20—C19—H19	109.5
C5—C4—H4	120.6	O6—C20—C19	109.7 (3)
C3—C4—H4	120.6	O6—C20—H20A	109.7
C4—C5—C6	122.1 (4)	C19—C20—H20A	109.7
C4—C5—H5A	119.0	O6—C20—H20B	109.7
C6—C5—H5A	119.0	C19—C20—H20B	109.7
C5—C6—C1	118.4 (4)	H20A—C20—H20B	108.2
C5—C6—C7	118.6 (4)	O7—C21—H21A	109.5
C1—C6—C7	123.0 (3)	O7—C21—H21B	109.5
N1—C7—C6	125.3 (4)	H21A—C21—H21B	109.5
N1—C7—H7	117.4	O7—C21—H21C	109.5
C6—C7—H7	117.4	H21A—C21—H21C	109.5
N1—C8—C9	109.1 (3)	H21B—C21—H21C	109.5
N1—C8—H8A	109.9	C21—O7—H7A	109.5
C9—C8—H8A	109.9		

### Hydrogen-bond geometry (Å, °)

**Table:**

*D*—H···*A*	*D*—H	H···*A*	*D*···*A*	*D*—H···*A*
O2—H2···O4	0.82	2.29	2.892 (4)	131
O3—H3···O7^i^	0.82	2.05	2.817 (6)	156
O3—H3···O6^i^	0.82	2.60	3.118 (6)	122
O5—H5···O7	0.82	1.88	2.567 (3)	141
O6—H6···O4^ii^	0.82	2.09	2.906 (4)	171
O7—H7A···O1	0.82	1.79	2.613 (5)	177

Symmetry codes: (i) *x*+1/2, *y*, −*z*+1/2; (ii) *x*−1/2, *y*, −*z*+1/2.
